# Facilitators and Barriers to the Implementation of Preschool Oral Healthcare Programme in Malaysia from the Perspective of Dental Therapists: A Qualitative Study

**DOI:** 10.3390/children7120266

**Published:** 2020-12-02

**Authors:** Muhammad Farid Nurdin, Zamros Yuzadi Mohd Yusof

**Affiliations:** 1 Oral Health Program, Ministry of Health, Level 5, Block E10, Precinct 1, Putrajaya 62590, Malaysia; mfaridnurdin@gmail.com; 2 Department of Community Oral Health & Clinical Prevention, Faculty of Dentistry, University of Malaya, Kuala Lumpur 50603, Malaysia

**Keywords:** preschool children, oral health, evaluation, dental therapists, Malaysia

## Abstract

The Preschool Oral Healthcare Programme (POHP) was introduced in Malaysia by the Ministry of Health in 1984 to provide oral healthcare for 5–6-year-old children. Most of its evaluations were directed towards assessing children’s oral health status. Little emphasis has been placed on assessing the programme feasibility from the perspectives of the oral health personnel. The objective of the study was to explore the facilitators and barriers to the implementation of the POHP using the perspectives of dental therapists (DT) in Selangor state, Malaysia. This study took a qualitative approach using focus group discussion (FGD) as the data collection method. The data were transcribed verbatim followed by thematic analysis using NVivo 12 Pro version software. A total of 13 FGDs had been conducted involving 114 DT. The main facilitators were good dental teamwork, assistance from schools and teachers, sufficient training of DT, adequate support from dental administration, and good cooperation from the children. The main barriers were lack of financial support, manpower, time, inadequate support from preschools and children, language barrier, and accessibility to sugary food and drinks at schools. The study provided important insights regarding the POHP that would be useful for programme improvement through policy changes, workforce training, and enhanced school participation.

## 1. Introduction

In 1984, the Ministry of Health (MOH) Malaysia introduced the preschool oral healthcare programme (POHP) with the objective of improving the oral health status of 5–6-year-old preschool children. This programme primarily focuses on oral health education (OHE) and clinical prevention at preschool centres. In 2003, the MOH published guidelines on oral healthcare for preschool children. In the guidelines, a few components of the POHP were revised including empowering dental therapists (DT) and teachers in school health and improving the oral health activities at preschool centres [1].

There are two components of the POHP. The first component involves two yearly visits to government preschools by DT. During the visits, DT will conduct oral health examinations, deliver OHE through puppets and songs, and supervise a tooth brushing drill (TBD) with the children. The second visit takes place after six months. It involves DT applying a fluoride varnish (FV) with 23,000 parts-per-million (ppm) fluoride on the children’s teeth, fissure sealants (FS), and doing simple treatment using glass ionomer restorations. The second component involves DT delivering an oral health seminar to preschool teachers once a year. It consists of OHE for children, an oral exhibition, a tooth brushing demonstration, and the distribution of OHE materials in the form of pamphlets, booklets, or soft copies [2].

Despite the implementation of POHP for over three decades, caries prevalence in this age group remains high. In the state of Selangor where the study was conducted, caries prevalence of 5-year olds in 2015 was 61.1% [3]. This prevalence was higher than the 50% target in the National Oral Health Plan (NOHP) 2011–2020 [4]. In addition, only 38.4% had no dental plaque and this percentage was lower than the national average of 40% [3].

Most evaluations of the POHP were quantitative in nature, i.e., percentage of coverage annually, the number of OHE sessions, fluoride varnish application and FS applied, treatment need estimation and assessment of caries and gingival health. So far, no studies have been performed to assess the implementation of the POHP [5]. An evaluation process using DT’s feedback is important because it allows the assessors to investigate if the POHP is carried out according to guidelines and to identify potential barriers. The findings could be useful to improve programme delivery, promote better outcomes, and to provide policymakers with vital information to support policy change if required [6].

As a result, this study aimed to evaluate the POHP using the perspectives of DT on the facilitators and barriers to implementing the programme in the state of Selangor.

## 2. Materials and Methods

### 2.1. Study Design

This was a qualitative study involving DT in the state of Selangor using focus group discussion (FGD) as the method of data collection [7].

### 2.2. Study Setting

The state of Selangor was chosen to be the study area due to several factors. First, it is one of the largest states in Malaysia with a mixture of urban and rural districts. In addition, differences in household income and parents’ education levels between districts provide a unique experience for DT in dealing with children from different socioeconomic backgrounds. Finally, the different types of government preschools in the urban and rural districts provide DT with a range of challenges in dealing with the different types of preschool administrations, facilities, and teachers. All these factors have major influences on the work experience, expectations, achievements, and the extent to which the POHP has been implemented according to the guidelines throughout the state. These factors enabled thorough discussions of the POHP among the DT.

### 2.3. Sampling and Recruitment

The study population included DT working in the state of Selangor. The inclusion criteria included DT with at least one year of work experience in the POHP. DT who fulfilled the inclusion criteria in the nine districts in Selangor were randomly selected to participate in the FGD. DT who held higher positions, i.e., sister or matron were grouped together. This ensured discussions among participants went smoothly without distractions by job positions in the service.

### 2.4. Development of FGD Questions and Training of Facilitators

A series of discussions were held between the researchers, dental public health specialists (DPHS), and dental administrators on the development of FGD main and probing questions. Subsequently, training of the FGD facilitator (FN) was conducted by a DPHS to ensure standardisation in the questions asked and probing for answers by the facilitator. Prior to data collection, a semi-structured interview schedule that contained the topic guide and probing questions was developed and piloted with a group of DT that was not involved in the main study to test the utility of the topic guide and the feasibility of FGD in field conditions [8]. The topic guides are shown in Table 1.

### 2.5. Conduct of FGDs, Data Analysis and Ethics

Each FGD consisted of 8 to 10 participants and was conducted in a separate room in each district’s main dental clinic. The sessions were facilitated by the researcher (FN) and were recorded using an audio recording tape in English and/or Malay language depending on the preference of the participants. A note-taker was appointed to take notes of important points from the discussion, observe the group dynamic, and to help the researcher in the organisation of the FGD.

The FGD began with an ice-breaking session between the facilitator and the participants. Then, the facilitator explained the objectives of the FGD and set out ground rules to be adhered to by all participants. When all participants had understood the rules, the facilitator started the FGD by asking the first question to the participants, i.e., “In your opinion, what are the factors that help or facilitate you in carrying out your duties in the POHP?” The participants were given enough time to discuss the question thoroughly and contribute their opinions on the topic with guidance from the facilitator until the discussion reached a saturation point whereby no new points were discussed. The probing questions were asked by the facilitator during the discussion to elicit detailed information from the participants if required and to direct the discussion to the main question. Next, the facilitator asked the second question, i.e., “In your opinion, what are the barriers that you face in carrying out your duties in the POHP?” This similar discussion process was repeated. Each FGD lasted between 60 to 90 min. At the end of the session, the facilitator summarised the main points and asked the participants if they wished to add any additional points. The FGD was concluded by the facilitator thanking the participants for their involvement in the FGD. The FGD was continued with different groups until the data obtained had reached saturation whereby further FGD did not produce new answers to the FGD questions.

Data analysis was carried out following the sequences by Gale et al. (2013). First, the audio recording from the interview was transcribed verbatim. This was followed by an open coding process and clustering of similar codes into themes [9]. The thematic analysis of the codes was done using NVivo 12 Pro version software.

In this study, rigour in research and data analysis was ensured by undertaking several steps, namely pilot testing of FGD, data saturation, triangulation, member checking, and negative case analysis [10]. Pilot testing was conducted before the actual FGD with 5 DT from Selangor to determine whether the questions asked were relevant and resulted in the right discussions as intended. Data saturation was achieved when no new information emerged from the discussions after several FGD sessions had been conducted. As mentioned earlier, the FGD sessions were recorded using voice recorder and there was a note-taker present to take note and observe the environment. This served as a triangulation of data from audiotape, notes, and observations during the FGD sessions [10].

After data analysis, a member check process was carried out with the participants. The transcripts from the FGD sessions were emailed to the participants to obtain their feedback. This was done to give them an opportunity to correct or challenge what they perceived differently from the transcriber interpretations. At the end of the data analysis, the findings were presented to the representatives of each district in a meeting to confirm the findings. This was also done for negative case analysis where the participants explained any discrepancies in the interpretations to avoid bias from the view of the researcher [10]. The investigator triangulation was performed by two investigators other than the main investigator [11]. These individuals were a DPHS and a layman. Several discussions were conducted to decide whether the interpretation and themes emerging from the verbatim were justified. This was done to minimise bias from the viewpoint of only one investigator. This process allowed multiple perspectives to be captured and more meaningful data generated [12].

Ethical approval for the study was obtained from the Medical Ethics Committee, University of Malaya [DF C01912/0054(P)]. Permission to conduct the study was given by the Oral Health Programme (OHP), Ministry of Health Malaysia and Oral Health Division (OHD) of Selangor [KKM.600-56/7/2 Jld.5 (91)]. Written informed consent was obtained from the participants prior to FGD.

## 3. Results

A total of 13 FGD sessions was conducted involving 114 DT from 9 districts in Selangor (Table 2). All participants were females and the majority were of Malay ethnicity. More than half worked in urban areas with a high percentage from the Gombak and Hulu Langat districts. The majority belonged to the 25–29 and 35–39-year-old age groups. The majority had up to 10 years of work experience.

### 3.1. Themes

**Figure 1:**
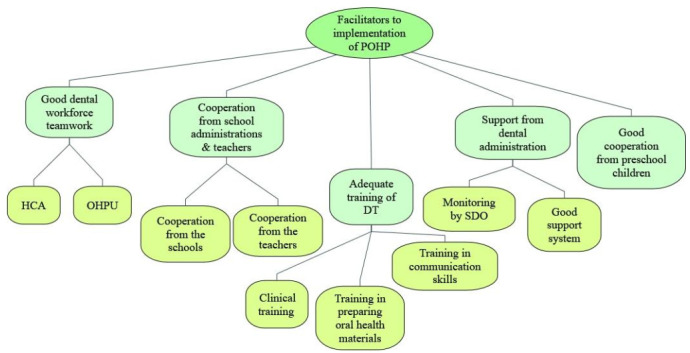
Facilitators to the implementation of preschool oral healthcare programme in Selangor state from the perspectives of dental therapists (POHP = preschool oral healthcare programme, HCA = healthcare assistants, OHPU = Oral Health Promotion Unit, DT = dental therapists, SDO = senior dental officers).

**Figure 2:**
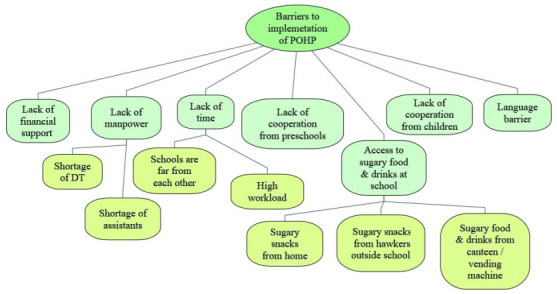
Barriers to the implementation of preschool oral healthcare programme in Selangor state from the perspectives of dental therapists (POHP = preschool oral healthcare programme, DT = dental therapists).

A total of five themes were identified as facilitators (Figure 1) while seven themes were identified as barriers to the implementation of the POHP in Selangor from the perspective of the DT (Figure 2).

#### 3.1.1. Facilitators to the Implementation of the POHP

##### Good Dental Workforce Teamwork

The teamwork among dental personnel was very good. The healthcare assistants (HCA) are well trained in helping DT to provide dental treatment to the children such as atraumatic restorative treatment (ART). In 2018, the OHD of Selangor established the Oral Health Promotion Unit (OHPU) for each dental clinic. This unit consists of two dental officers (DO) and one dental therapist that aims to provide oral health education (OHE) such as TBD to all preschools. The OHPU helps in reducing the time taken during each visit as they provide OHE while DT concentrates on providing dental treatment.

*“…OHPU helps in reducing time, as OHPU can concentrate on giving OHE and TBD, and the dental mobile team (DT, HCA) for preschool children can work on the clinical aspect of the visit”*

(DT 6, Gombak 2nd group)

The OHPU also led to a better perception towards the dental team by the teachers as there were dental officers in the unit.

*“Teachers would be delighted when OHPU came to schools because they are dentists in the unit. The teachers’ attention towards the DO was different compared to us (DT) who wear white uniform. They do not really give full attention to us”*

(DT 4, Klang 2nd group)

It was also reported that the cooperation between dental personnel was very good. They were willing to cover each other’s schedule if one person attended a course or had to take emergency leave.

*“Between DT, the cooperation is good, we are really close and very positive. We help each other if one has to go for a course or has to take emergency leave”*

(DT 7, Petaling)

##### Cooperation from School Administrations and Teachers

The DT commented that the school administrations were quite accepting towards the POHP now compared to a few years ago. They received good cooperation from preschools during their visits. Even the top management showed interest in the dental treatment that the children received.

*“Sometimes the headmaster would also come and monitor our programme. If he coincidently walks by our treatment room, he will look inside, at the children having the treatment and he would also like to know about the treatment”*

(DT 10, Hulu Selangor)

The school administrations also helped to reserve the date for the dental team to come to the school after being informed by the dental clinic. Additionally, some of the preschools even organised an oral health corner upon receiving advice from the OHPU. This showed that the preschool administrations were accepting of the programme and were willing to cooperate with and take advice from the dental team.

*“Let say if we give them appointment date, they will reserve the date only for us”*

(DT 2, Hulu Langat 2nd group).

*“The OHPU will encourage the preschool to prepare an oral health corner, and some preschools took up the advice and prepared a suitable oral health corner (as suggested by the dental team)”*

(DT 2, Klang 1st group)

The preschool teachers would also call the dental team prior to their visit and inquire if the dental team needed anything so that they could prepare.

*“The teachers asked us nicely what we needed before we came for our visit. I informed the teachers (that) we would come the next day, please ask the preschool children to bring their toothbrush. The message will surely reach the children”*

(DT 6, Sepang).

Consent from parents is required before a dental check-up and treatment could be done on preschool children. The teachers would act as the intermediary between the dental team and the parents. The teachers also ensured that parents were informed about the dental visit and that parents would bring their children to the school on the visiting day of the dental team.

*“We informed the teachers about our visit and asked them to remind the parents, so that all the children will be there during our visit”*

(DT 9, Hulu Langat 2nd group)

During the visit, the preschool teachers helped to arrange the children for the activities such as TBD and OHE talk. The teachers were also eager to learn, especially the correct tooth brushing technique, so that they can apply the knowledge and skills with the children during daily tooth brushing activity and in games or quizzes.

*“We can see that the children are ready, the teachers already arranged the children. They knew that we are coming, and the children are in orderly manner”*

(DT 3, Petaling)

Some of the preschool children were uncooperative. Nevertheless, the teachers helped the DT to calm the children down throughout the treatment.

*“If the preschool children were uncooperative during the treatment, the teachers would wait by their side and hold the children’s hands until the treatment are completed”*

(DT 10, Kuala Selangor)

##### Adequate Training of DT

The training that the DT received at dental college was very helpful in preparing them for handling the preschool children. The DT commented that the training they received was divided into handling, communicating, and treating preschool children as well as producing OHE materials. They were also trained in clinical prevention such as applying fluoride varnish and placing fissure sealants on children’s teeth.

*“At dental college, we learned how to handle preschool children. There were preschool children invited to the college. It helped a lot”*

(DT 3, Gombak 1st group)

For OHE materials, the DT were taught how to produce the materials from the beginning. This helped them prepare OHE materials when they started working.

*“During our training at the college, we were given a lecture on how to prepare slides show suitable for preschool children”*

(DT 6, Sabak Bernam)

*“We were also trained how to make dolls, puppets, and how to conduct tooth brushing drills. We sat for an examination in oral health promotion”*

(DT 8, Petaling)

Continuous education for DT is essential to update them with the current materials and methods used in OHE for preschool children and how to make them. To assist them, various training courses are provided from time to time.

*“There are courses such as how to conduct OHE using puppets. It is a 3-day course. The last time such a course was held was when I first joined the service 5 years ago. It involved all districts in Selangor state with invited speakers came from outside the dental service”*

(DT 9, Gombak 1st group)

The state and district oral health divisions also provide courses in communication skills for dental personnel and hands-on training for handling preschool children.

*“The latest course was on how to handle children and communicate with children. The lecturer was not from dental (outsource). He is a specialist from Teachers Learning Institute (TLI). Many speakers were involved. A puppet specialist taught us how to do voice intonation”*

(DT 6, Hulu Langat 1st group)

##### Support from Dental Administration

The DT commented that the support they received from the dental administration from the state OHD and district senior dental officers (SDO) was crucial in assisting them to implement the POHP. The OHPU that was created by the state OHD to assist DT in OHE of preschool children was very helpful.

*“The SDO gives full support by asking a dental officer to be involved in the POHP through the OHPU. The dental officer will be in the same team with the dental therapist and helps in OHE”*

(DT 10, Gombak group 2)

The state OHD also ensured that replacement staff were available if any of the personnel were on leave or had to go for a course.

*“Our dental officer in-charge would ask a dentist who is free to replace the DT who could not go to the preschools”*

(DT6, Hulu Langat 2nd group)

The DT explained that the state OHD and district SDO regularly monitored the activities and treatment provision to the children. This was done either by the SDO themselves or through the dental officer in charge of the clinic, a sister, or a matron.

*“The SDO really put an emphasis on the fluoride varnish application to the children because it is one of our core duties. She would remind us to get it done quickly. She would also ask if we faced with any problem, or if we want to add more preschool centres in the schedule”*

(DT 6, Gombak 1st group)

##### Good Cooperation from Preschool Children

The DT commented that most preschool children were willing to cooperate during dental treatment and this had resulted in the programme running smoothly according to the schedule.

*“Children received a lot of dental exposures nowadays. Most children are not afraid of dental treatment anymore and they are ready. From that angle it is obvious”*

(DT 1, Petaling)

*“During our visit to the preschool centre, when we called the children for dental check-up, they did not cry. The teacher did not need to tell them to sit on the chair and open their mouth. They were doing it willingly”*

(DT 4, Hulu Langat 2nd group)

#### 3.1.2. Barriers to the Implementation of the POHP

##### Lack of Financial Support

The DT stated that there was a lack of financial support for developing new OHE materials. They commented further that occasionally the dental officer in charge had to use his own money to purchase new OHE materials. Although he was allowed to claim back the money he had spent, it would take a long time for the reimbursement of expenses. As a result, most of the OHE materials were often reused several times over a few years. Consequently, many children lost interest as the activities were repeated.

*“If we keep bringing the same materials every year, the children will get bored, so we need to make or purchase new materials. But these require money, so we have little choice except to use our own money as there was no budget for it. Sometimes, the teachers ask for posters to put on the wall but there are no extra posters”*

(DT 7, Hulu Langat 2nd group)

*“In the plan of action, we need to produce at least one OHE material per year, but there is no funding provided. So, we had little choice except to collect money among us, or we tried to construct something. We have to comply with the plan of action, and that means we need to have at least one OHE material for each clinic, every year”*

(DT 1, Gombak 2nd group)

##### Lack of Manpower

The participants agreed that there was a shortage of DT that resulted in the increased workload of the current DT. Consequently, this might affect the quality of work and motivation among the DT. Although the POHP in Selangor mainly focuses on government preschools, the programme is also extended to private preschools registered under the Department of Education, Selangor upon request from the preschools. In addition, the SDO will include one additional preschool to be visited by the DT team each year. This results in the DT having a heavier workload each year.

*“…the number of preschools (need to be covered) is increasing. We have difficulties in visiting all of the preschool centres with the shortage of manpower. Also, the number of staff is small. There is no replacement staff for those who have retired or transferred out”*

(DT 3, Sepang)

Although the establishment of the OHPU was seen to be the facilitating factor in some districts, some DT disagreed as they viewed the OHPU as having the same job scope as them. Some DT commented that there was no distinctive job scope for the OHPU personnel and that the activities carried out by them were also performed by the DT team.

*“They (the OHPU) were not given any guidelines on what to do. They just continue the work like the previous year which was also conducted by DT”*

(DT 9, Klang 2nd group)

*“The earlier agreement was to replace the DT in the OHPU with a DO to go to school. However, this has not happened as we are also short of DO for the POHP”*

(DT 8, Kuala Selangor)

Some of the DT commented that although a few districts had a sufficient number of DT, there was a shortage of dental assistants. The DT stated that it was very difficult for them to perform treatments at preschools without dental assistants assisting them.

*“Sometimes, we were not given any assistant although we needed it. We need a dental assistant to assist us in the treatment of the preschool children. We had an appointment with the teacher to come to the school. But when we did not have an assistant, or if we were denied an assistant, we had to cancel our visit to the school”*

(DT 9, Kuala Selangor).

##### Lack of Time

The majority of DT commented that the increased number of preschool centres, the increased number of enrolments, and the shortage of manpower had led to DT having less time to complete their work. The DT commented further that sometimes they had to visit 2 to 3 preschools a day and that they felt exhausted. On more than one occasion when they arrived at the preschools, the children were having their morning break. Subsequently, they had to wait until the children finished their meal.

*“…sometimes the schedule was very pack, had to cover 2 or 3 preschool per day”*

(DT 7, Kuala Langat)

*“Sometimes, we supposed to go to 2 preschools inside the school premise, and 2 kindergartens. However, we could cover only one preschool. There was no time to visit all. When this happened, it disturbed our schedule for the week”*

(DT 6, Sabak Bernam)

In some districts, the preschools are far from each other, especially in rural areas. As a result, the DT team took a long time to reach the preschools. Some preschools required half a day to visit because of their remote locations.

*“The location between preschools are not near to each other. Moreover, on Friday they will be a lot of movement, some DT would go for courses, some would go to secondary schools”*

(DT 3, Kuala Selangor)

##### Lack of Cooperation from Preschools

Many DT mentioned that a few preschool administrators were uncooperative and demanding. Some of the preschools refused to accept the visiting dates allocated to them. This usually happens with private preschools.

*“The schools did not cooperate with us. We must follow their request. We have a lot of preschools to visit. So, we informed them of our visiting date much earlier, but they refused to accept it, they wanted us to follow their schedule”*

(DT 5, Hulu Langat 1st group)

Not only did they refuse to follow the date given, but some of them did not want the second visit from the programme. Again, this usually happens with private preschool centres. This affected the running and effectiveness of the programme as a whole.

*“They only want the first visit. They always refuse when we informed them about the second visit. Their excuse was, our schedule is very pack”*

(Dental therapist 7, Klang 1st group)

The school administration was also very strict with the timing of the programme. It was reported that there was an incident where the headmaster came and stopped the programme halfway as it had exceeded the time allocated.

*“…the staff was in the middle of giving OHE using a video presentation when the headmaster came and switch off the LCD, he said the children had enough with the talk and should do tooth brushing exercise. He dictated the programme, and we must follow the school’s regulations”*

(DT 5, Hulu Langat 1st group)

##### Lack of Cooperation from Children

As the POHP targets preschool children, lack of cooperation from them had invariably affected the implementation of this programme. It was reported that the DT occasionally had a hard time handling preschool children in large groups. Some of them would stand up and make noise. Some showed no interest because they had known about the talk and activities conducted by the DT from last year’s visit.

*“The children can be hard to handle, especially when there were many of them. Some of them would stand up and sit down repeatedly”*

(DT 3, Kuala Selangor)

*“The children were bored, every year it is the same thing. Those who attended the preschool at 4 years old had similar activities when they were 5 and 6 years old”*

(DT 3, Sepang)

Dental fear was a normal occurrence during DT’s visit to the preschools. Some of the children were afraid of the white uniform worn by the DT and subsequently refused to take part in the activities.

*“…they were afraid of the white uniform”*

(DT 6, Gombak group)

*“Some children cried because they were too afraid when they saw us and they refused treatment”*

(DT, Hulu Selangor)

##### Language Barrier

In a multiracial country like Malaysia, children speak various languages at home according to their ethnicities and social background. As a result, many children in preschools had mother tongues other than the national language. Typically, it is hard for preschool children to understand other languages except for their mother tongue. As all the activities conducted by DT were delivered in the Malay (national language) or English language (second language), many of the children could not fully understand the instructions given. As a result, the activities conducted were not fully understood by the children except for skills-based activities such as tooth brushing exercise.

*“In Chinese schools, the teachers would translate for us. It was a bit difficult if we want to communicate directly with the children”*

(DT 9, Gombak 1st group)

*“In preschools where there were many children who speak Tamil, the teacher had to explain to the children the messages that we delivered to the children”*

(DT 5, Hulu Selangor)

*“But when we went to the aborigine’s area, they appeared to be lost. I mean, do they understand what we are talking about? The teachers would translate and explain to the children in their language”*

(DT 7, Sepang)

##### Accessibility to Sugary Food and Drinks at School

The DT commented that although there are guidelines on food preparation for preschools introduced by the MOH, some preschools did not follow the guidelines, in particular the private preschools. To ensure the preschool environment is a conducive place to instill good eating habits, sugary food and drinks must be avoided. If the teachers still provide sugary food and drinks, the children might think it is appropriate for them to consume it as it was given by their teachers.

The teachers were instructed by the preschool administration to buy any type of food that they could get for the children including sugary food such as cakes. Some children brought sugary food and carbonated drinks to school and the teachers allowed this.

*“…some private preschools have their own menu, some cook at the centre (at the preschool), some allowed children to bring food from home. I’ve seen that children who brought food from home would bring junk food, packet drinks, and carbonated drinks that are not healthy”*

(DT 6, Sabak Bernam)

Children from preschools that are located inside primary schools often buy their food from the school canteen. Although the school canteen must follow the guidelines from the MOH, some canteens continue to sell sugary food such as doughnuts and ice cream. There were also vending machines inside the school premises that sold carbonated drinks. This gave a wrong impression to the children that they can take sugary food and drinks at school. The children also purchased their sugary food and drinks from vendors and shops outside the school premises.

*“The primary school has a canteen. The canteen sells sugary food such as doughnut and ice cream. The school also has a vending machine selling carbonated drinks”*

(DT 5, Klang 1st group)

*“Most of the children bought junk food from outside the school premise. This contributed to caries”*

(DT 2, Kuala Selangor)

## 4. Discussion

This study aimed to explore the facilitators and barriers of implementing the POHP in Selangor state from the perspective of DT. Teamwork within the team and the formation of OHPU were the main facilitating factors of implementing the POHP. Although some of the DT found OHPU as a barrier of implementation due to redundant job scopes, the majority found it as a factor that could reduce time, and they could concentrate on the clinical aspects of the visit. Approaches towards prevention would not be successful without complete involvement from every personnel. Teamwork in prevention activities would take time, but it offers a basis for enhanced health outcomes [13]. The DT who perceived OHPU as a barrier may not be clear about the objectives of forming the OHPU. Better communication between the administration and staff on the job descriptions of OHPU could help prevent confusion. The need to improve staff role descriptions, job descriptions, performance assessment, services objectives, and communications and relations between staff was associated with poor comprehension of the programme mission [14].

Despite the good teamwork among the implementers, the shortage of manpower was one of the major issues hindering the progress of the programme. This situation was also faced in Australia when providing dental services to remote Aboriginal communities. The dental workforce shortage heavily impacted the public sectors, especially in rural areas [15]. Although POHP in Selangor also involved both urban and rural areas, the impact was similar to the situation in Australia. To increase the number of OH personnel would incur a higher cost for the government. It was reported that from the total expenditure of the programme, 45% of the total cost was for manpower, and this amount omitted management staff [16].

The acceptance and reactions of the school administration, teachers, and children towards the POHP played an essential role in its implementation. A study in Thailand stated that the caregivers were thankful and happy that an OH promotion team visited to help improve their child’s oral health. However, the staff were unsure whether the caregivers really did follow the recommendations [17]. This is relatable to POHP as the teachers were very helpful and happy during the visit. However, to conduct the activities suggested by the dental team, it is up to the teachers’ initiative. A study carried out in Grenada also revealed a similar situation where more than 70% of the teachers accepted that a 2-min brushing routine at school was not disturbing the class and accepted the school-based programme extremely well [18].

Getting consent for examination and treatment from parents was an essential process prior to conducting the POHP. The teachers played an important role in facilitating the DT in this aspect. Not getting consent would disrupt the implementation of POHP. This agrees with a study done in London where the authors revealed that some schools struggled with the return of consent forms, despite being rushed by the Dental Public Health (DPH) team [19].

Support from the dental administration, either financial or motivational is an essential component of every oral health programme. Providing further training on management of preschool children helps to facilitate the programme. A study in Sydney, Australia on family health nurses promoting an oral healthcare programme reported that the nurses were satisfied with the training and resources provided for the early childhood oral healthcare programme. However, some of them suggested that the training should be conducted at least once a year [20]. This sentiment was like the response from the DT in Selangor where they suggested more training should be conducted on how to handle and treat preschool children.

Another factor that highly influences the implementation of an oral health programme is financial support. It was described that children with untreated early childhood caries (ECC) are more likely to reside in an area with lower healthcare expenditure per capita, compared to children with treated ECC [21]. It shows that to treat dental diseases, optimal financial support should be allocated. In Malaysia, the POHP was fully supported financially by the government. Due to budget constraints, the DT had difficulties in providing updated OH promotional materials. Insufficient funds and incentives decreased the motivations of the OH personnel to conduct the school-based prevention programmes [22]. However, in New Mexico, additional funding for school-based oral healthcare programmes could be requested from the state senators (for a limited amount). Although the amount could not cover the cost of the programme, it could aid in obtaining dental materials and equipment [23].

Updated OH promotional materials are also very important to keep children interested. Nowadays, young children are exposed to technology early at home by their parents or older siblings. A study involving children aged 3–5 years stated that engagement with technology at home was influenced by parents and older siblings, via multiple ways of interactions [24]. OH education could be given using computers or tablets to ensure the children are interested to listen to the DT. Education could be organised according to children’s capacity, knowledge, learning pace, and individual necessities using computers. Integrating computers into education could also positively influence children’s social-affective development, psychomotor skills, cognitive and language abilities, and would not obstruct cooperation and communication [25].

Children’s acceptance and reactions towards the programme may contribute to the flow of the implementation. Dental fear and anxiety among adults and children have been documented as a problem in dentistry for numerous years. It is reported that dental fear and anxiety affect 10% of the population. However, it decreases with age. Dental fear and anxiety are also reported to affect girls more and are associated with dental pain [26]. It would be very difficult for the DT to carry out the procedures if the child refuses to cooperate. A fluoride varnish application programme in Pahang, Malaysia also reported that the procedure (application of fluoride varnish) became difficult when the children refused to open their mouth and were being uncooperative [27]. The DT reported that most of the children who exhibit dental fear had caries. They were afraid that the DT would perform dental extraction. A study done in Australia showed that there were higher levels of dental fear in children with dental caries and pain, irrespective of the mothers’ oral health and family socio-demographic status [26]. Another study also stated that children’s dental fear was associated with their general fear but not maternal dental fear [28]. However, a systematic review stated that although dental fear is associated with dental caries, children with low family income also had higher dental fear [29].

The language used in oral health promotion is very important in ensuring the children receive the messages clearly. As reported earlier, some children were unable to understand the national language as they speak their mother tongue at home. Moreover, the private preschools in Malaysia permit the use of any language other than Malay (National Language) as the medium of instruction for teaching the standardised national curriculum. These languages can be Chinese, Tamil, or English [30]. A study done in Olathe, Kansas, USA revealed that language was also one of the barriers to extending oral healthcare services in school, apart from cost and lack of providers [31]. To overcome this issue, diversifying the oral healthcare workforce to cater to all ethnicities should be considered [32].

It is very difficult for the DT to advocate a healthy diet when a sugary diet could easily be obtained at school. Food sold at the canteen in primary schools were rich in carbohydrates, high in fat, and contained added sugars [33]. This also includes sugary food brought from home during parties or celebrations. The same situation happened in the United States of America, where less than 10% of schools proscribed sugary diets during classroom parties [34]. All these factors could obstruct the effort done by the dental team, as it is evident that early childhood caries (ECC) is associated with sugary food consumption among preschool children. Tooth brushing could weaken the association but only to a certain extent, and not totally [35].

Implementation of oral healthcare programme from an early age to instill good oral health behaviours is vital. This is because healthy oral health habits developed at a young age tend to last until adulthood. A study on third-year dental students to instill the usage of interdental brushing revealed that it was challenging to introduce a new habit in adults when they have been practicing tooth brushing only since childhood [36]. Furthermore, a less intensified intervention when implemented early in life was comparable to a more intensified intervention started at a later age [37]. Therefore, the POHP implementation in Malaysia is the right step for developing good oral health behaviours in adults.

This study has several limitations. First, all participants in this study were females. This is because only females are accepted to study Diploma in Dental Therapist in Malaysia [38]. Hence, no male participant was recruited. Second, a phenomenon called the Hawthorne effect could happen during the interview as the interviewer was an oral health personnel from the MOH [39]. However, the participants were informed that their input was confidential and no individual comment could be identified from the study. As the study was conducted in Selangor state, the barriers and facilitators might not be similar to other states in Malaysia, especially East Malaysia (Sabah and Sarawak).

## 5. Conclusions

Despite the POHP being a well-established programme in Malaysia, these findings provide important insights into the facilitators and barriers to the implementation of POHP in Malaysia from the perspective of DT. The findings are useful to guide policymakers and stakeholders to improve the POHP through policy changes, financial aid, human resource development, and enhanced school cooperation.

## Figures and Tables

**Table 1:** Topic guides of the Focus Group Discussion.

No	Leading Question	Probing Question
**1.**	Introduction Can you briefly explain your roles in the POHP in Selangor?	Not applicable
**2.**	Feasibility A. Facilitating factors i.In your opinion, what are the factors that help or facilitate you in carrying out your duties in the POHP?	(a) Children’s reactions
		(b) Dental administration’s factors
		(c) Workforce
		(d) Dental material/financial aid/transportation
		(e) Preschool centre administration/teachers’ factors
		(f) Training
		(g) Time factors
	B. Barriers ii.In your opinion, what are the barriers that you face in carrying out your duties in the POHP?	(h) Children’s reactions
		(i) Dental administration’s factors
		(j) Workforce
		(k) Dental material/financial aid/transportation
		(l) Preschool centre administration/teachers’ factors
		(m) Training
		(n) Time factors

**Table 2:** Socio-demographic profile of the dental therapists (*n* = 114).

Variable	*n*(%)
**District**	
**Gombak**	21(18.4)
**Hulu Selangor**	11(9.6)
**Hulu Langat**	21(18.4)
**Klang**	17(14.9)
**Kuala Langat**	8(7.0)
**Kuala Selangor**	13(11.4)
**Petaling**	8(7.0)
**Sabak Bernam**	8(7.0)
**Sepang**	7(6.1)
**Area**	
**Urban**	68(59.6)
**Rural**	46(40.4)
**Race**	
**Malay**	99(86.8)
**Chinese**	1(0.9)
**Indian**	4(3.5)
**Other**	10(8.8)
**Age/year ^1^**	
**20–24**	14(12.3)
**25–29**	32(28.1)
**30–34**	10(8.8)
**35–39**	34(29.8)
**40–44**	12(10.5)
**45–49**	7(6.1)
**50–54**	1(0.9)
**Working experience/year ^1^**	
**1–4**	34(29.8)
**5–9**	21(18.4)
**10–14**	21(18.4)
**15–19**	24(21.1)
**20–24**	7(6.10
**24–29**	2(1.8)
**≥ 30**	4(3.5)

^1^*n* not equal to 114 due to missing data.
